# CO_2_-Responsive Vinyl Polymers: From Synthesis to Application

**DOI:** 10.3390/molecules30112350

**Published:** 2025-05-28

**Authors:** Mahshab Sheraz, Rui Wang

**Affiliations:** School of Environmental Science and Engineering, Shandong University, Qingdao 266237, China

**Keywords:** CO_2_-responsive polymers, advanced radical polymerization, functional group-induced transitions, reversible polymer behaviors, smart applications

## Abstract

CO_2_-responsive polymers have emerged as a significant class of smart materials, distinguished by their ability to reversibly alter their properties upon exposure to CO_2_. Due to CO_2_’s abundant availability, low cost, non-toxicity, energy efficiency, and excellent biocompatibility, these polymers offer remarkable environmental and practical advantages. This review succinctly explores recent advancements in the synthesis, mechanisms, and applications of CO_2_-responsive polymers, emphasizing the pivotal roles of specific acidic and basic functional groups such as carboxylic acids, phenolic groups, amines, amidines, guanidines, and imidazoles. Advanced polymerization techniques including free radical polymerization (FRP), atom transfer radical polymerization (ATRP), reversible addition-fragmentation chain transfer (RAFT), and nitroxide-mediated polymerization (NMP) are critically evaluated for their precision and flexibility in polymer design. Significant applications in smart separation, carbon capture, drug delivery, desalination, emulsions, tissue engineering, and sensing technologies are discussed comprehensively. Although substantial progress has been made, ongoing challenges include enhancing response speed, durability, sustainability, and economic viability. Future research is recommended to focus on innovative polymer structures, computational modeling, hybrid materials, and greener synthesis methods. This review aims to inspire continued exploration and practical utilization of CO_2_-responsive polymers to address pressing environmental and technological needs.

## 1. Introduction

Stimuli-responsive materials demonstrate the capacity to react to external or internal stimuli through transitional behaviors, representing a significant area of interest within the field of smart systems [1,2,3,4]. Consequently, stimuli-responsive polymers have been receiving significant attention in recent years. Responsive polymers exhibit adaptive “smart” responses to subtle variations in environmental signals, including temperature [5,6], pH [7,8,9], light [10,11], ionic strength [12,13], enzymes [14,15,16], redox conditions [17,18], magnetic fields [19,20,21], and in mechanical forces [22,23]. The latest developments in the field of stimuli-responsive polymers offer potential applications in biomedical delivery [24], including drug and gene carriers and sensors, as well as in the development of “smart” surfaces, catalysts [25], and adhesives [26]. However, notable limitations are present in the application of these triggers, including economic and environmental costs, as well as product contamination, which render them less appealing for commercial use [27].

Notably, among these stimuli-sensitive polymeric systems, CO_2_ has garnered significant attention in recent years as a benign, inexpensive, abundant, and non-toxic trigger for stimuli-responsive materials. Because of the environmentally friendly attributes provided by the CO_2_ trigger, in comparison to the existing triggers, CO_2_ presents several distinct advantages [28,29,30,31,32,33,34].

Unlike temperature-responsive polymers that require heating or cooling to trigger changes, CO_2_-responsive polymers achieve transition behaviors simply through the addition or removal of CO_2_ gas, which alters the solution’s pH [28]. This approach is cost-effective due to CO_2_’s abundance and environmental friendliness, making it an increasingly attractive research topic [35,36]. Compared to pH-, ionic-, enzyme-, or redox-responsive polymers, which often require repeated chemical additions and produce by-products, CO_2_-responsive polymers enable reversible cycling by alternately purging CO_2_ and inert gases (argon or nitrogen) without contamination, maintaining high responsiveness over multiple cycles [37,38]. Moreover, because CO_2_ diffuses easily through water, it offers greater penetration depth than stimuli like light, magnetic fields, or mechanical forces, enabling deeper material responses. Finally, as a natural, biocompatible metabolite with excellent membrane permeability, CO_2_ enhances the biomedical potential of CO_2_-responsive polymers [35,39,40,41]. Therefore, CO_2_-responsive polymers have been extensively used in various applications, including oil–water separation systems [42,43], carbon capture and storage [44,45], drug delivery systems [45], forward osmosis desalination [46], tissue engineering [47], smart emulsions [48], CO_2_ sensing [49], and others [50].

CO_2_-responsive polymers can be synthesized by using different functional groups that enable reversible protonation, deprotonation, ionic interactions, or complexation in response to CO_2_ stimuli. These groups can be categorized into acidic moieties (such as carboxylic acids and phenols) and basic groups (including amines, amidines, guanidines, and imidazoles). They facilitate adjustable modifications in solubility, hydrophobicity, charge, and conformation, influenced by their type, distribution, and concentration [30]. Recent developments in synthetic methodologies, including free radical polymerization (FRP) [51], atom transfer radical polymerization (ATRP) [52], nitroxide-mediated polymerization (NMP) [53], and reversible addition-fragmentation chain transfer (RAFT) polymerization [54], have broadened the design possibilities for CO_2_-responsive polymers. Controlled and living radical polymerizations, such as ATRP, RAFT, and NMP, enable the precise customization of polymer architectures. This capability facilitates the synthesis of complex structures, including block copolymers and graft copolymers, which enhances their functional responsiveness and expands their range of applications.

Due to the numerous advantages of CO_2_ as a stimulus compared to other triggers, the number of publications on CO_2_-switchable (or CO_2_-responsive) materials has increased significantly in recent years. Although some review articles were published on this topic between 2015 and 2019, they are now considered outdated given the rapid advancements in this field [30,31,55]. Therefore, there remains a lack of a recent comprehensive review article specifically dedicated to CO_2_-responsive polymers. In particular, there is a need for a review that focuses on advanced synthetic methodologies and comparative analyses of different polymerization techniques, as well as a detailed exploration of their state-of-the-art applications across diverse technological domains. This review aims to fill that gap by providing a systematic and critical analysis of the latest developments, offering insights into current achievements, identifying prevailing challenges, and proposing future research directions. This work aspires to stimulate further innovation and guide ongoing efforts toward the full realization of the potential of CO_2_-responsive polymers in advanced applications.

## 2. CO_2_-Responsive Polymer Mechanisms

### 2.1. Acidic Functional Groups

**Figure 1:**
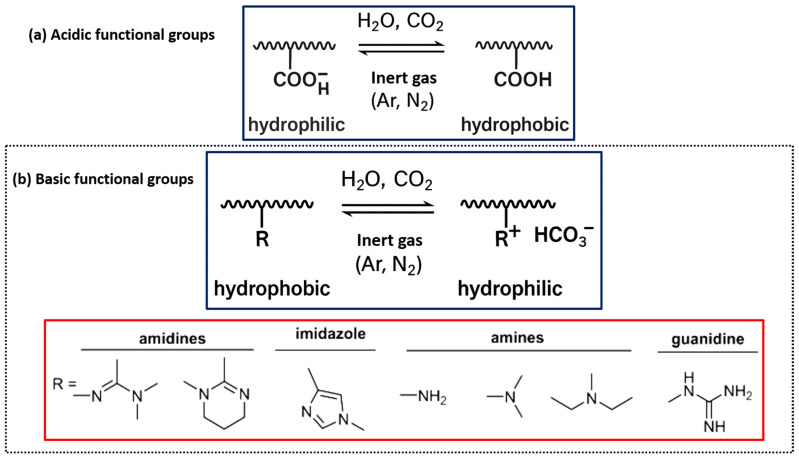
Various types of CO_2_-responsive polymers. (**a**) Acidic groups (e.g., carboxylic acids, phenols) and (**b**) basic groups (e.g., amines, imidazoles, amidines, guanidines) that undergo reversible changes under CO_2_ exposure.

This group was initially revealed by Moore and Lefevre, who showed that exposure to CO_2_ could trigger coagulation in latex resins stabilized by fatty acids [56]. Acidic functional groups endure modifications in their protonation state upon exposure to CO_2_, hence affecting the polymer’s charge and solubility characteristics [57]. The most acidic functional groups in CO_2_-responsive polymers are carboxylic acids and phenolic groups [58]. After contact with CO_2_, these groups become protonated, transitioning the polymer from a more hydrophobic to a hydrophilic state, as shown in Figure 1a [59].

Carboxylic acids, represented by poly(acrylic acid) (PAA) and poly(methacrylic acid) (PMAA), are the most deeply investigated CO_2_-responsive acidic functional groups. Carboxylic acids, in their neutral state, are weak acids that can deprotonate at boosted pH levels to yield carboxylate anions (–COO^−^) [60]. Exposure to CO_2_ triggers a decline in the solution’s pH caused by the generation of carbonic acid, which protonates the carboxylate group, enhancing its hydrophilicity. The change in charge enhances the polymer’s water solubility, perhaps resulting in modifications to the polymer’s swelling behavior and self-assembly characteristics [58,61].

### 2.2. Basic Functional Groups

Basic functional groups can generate stable salts with CO_2_, leading to increased hydrophilicity of the polymer [62]. The primary functional groups that confer CO_2_-responsiveness to polymers include amines, imidazoles, amidines, and guanidines, as illustrated in Figure 1b. These groups can collaborate with CO_2_ to produce ammonium salts, carbamates, or bicarbonates, leading to substantial alterations in the polymer’s characteristics [55].

#### 2.2.1. Amidines Groups

The CO_2_-responsive properties of amidine functional groups were first discovered by Jessop et al. (2006) [63], marking a significant advancement in the field of dynamic and switchable materials. Amidines are characterized by their relatively high basicity, which is stronger than that of amines but lower than guanidines, which enables efficient interaction with CO_2_ in aqueous environments [64]. In the absence of N–H bonds, amidines reversibly react with CO_2_ and water to form bicarbonate salts, which can subsequently regenerate the neutral amidine upon CO_2_ removal. However, when N–H bonds are present, the formation of carbamate salts may occur either alongside or instead of bicarbonates, altering the switching pathway [60].

One notable advantage of amidines is their faster conversion to bicarbonate salts compared to tertiary amines. However, the regeneration of the neutral form is a more thermodynamically demanding process owing to their higher basicity, making CO_2_ release slower and more energy-intensive [54]. The basicity and thus the CO_2_-responsiveness of amidines can be finely tuned through structural modifications, such as the incorporation of aromatic substituents on the nitrogen or central carbon atoms. These modifications lower the basicity, resulting in amidine systems with switching behavior more akin to tertiary amines [65]. Due to their rapid response to CO_2_ and structural tunability, amidines have emerged as promising candidates for incorporation into CO_2_-switchable (co)polymers, offering the potential for a wide range of applications in stimuli-responsive materials and environmentally adaptive systems [66].

#### 2.2.2. Imidazole Groups

Imidazole is a heterocyclic compound containing nitrogen atoms that is capable of reversible protonation when exposed to CO_2_. When exposed to CO_2_ in aqueous media, imidazole confronts protonation, resulting in the formation of imidazolium bicarbonate, which enhances the hydrophilicity of the polymer. This characteristic renders imidazole a flexible functional group in CO_2_-responsive systems. Polymers with imidazole groups show variations in solubility and require volume phase transitions during CO_2_–N_2_ cycling [67]. Microgels synthesized with 1-vinyl imidazole (VIM) demonstrate reversible swelling in water when exposed to CO_2_, attributed to protonation, and they contract upon treatment with N_2_. This behavior enhances applications in responsive soft materials and catalysis. Furthermore, imidazole-functionalized microgels have served as catalysts for the cycloaddition of CO_2_ to epoxides. Their capacity to capture CO_2_ and adjust catalytic activity under mild conditions emphasizes their dual function as both a responsive and functional group [54,67].

#### 2.2.3. Amino Groups

Amines are among the most prevalent basic functional groups for CO_2_-responsive polymers. Amines have lower basicity compared to alkylguanidines and alkylamidines. A prevalent criterion to determine basicity is the dissociation constant (p*K*_aH_) of the conjugate acid; a higher p*K*_aH_ indicates a more potent base [68]. Molecules bearing amine functionalities, such as tertiary amines and sterically hindered primary and secondary amines, are capable of interacting with CO_2_ in aqueous media to form the corresponding bicarbonate salts. The relatively mild basicity of tertiary amines facilitates enhanced reversibility in CO_2_ capture–release cycles, allowing for CO_2_ desorption at ambient temperature in contrast to primary and secondary amines, which require thermal input for effective CO_2_ release [69]. Although tertiary amines exhibit lower basicity compared to stronger organic bases such as amidines and guanidines, their resulting quaternary ammonium bicarbonate salts demonstrate superior hydrolytic stability following CO_2_ purging [70].

Tertiary amine-containing molecules demonstrate excellent ionic conversion efficiency in aqueous environments, facilitating complete separation within water–tetrahydrofuran (THF) cosolvent systems. However, their application at elevated temperatures is limited due to a decrease in percent protonation, which necessitates increased CO_2_ pressure to maintain effective switching behavior at higher temperatures [27]. For instance, Darabi et al. (2014) [71] explored nitroxide-mediated polymerization (NMP) of 2-(diethylamino)ethyl methacrylate (DEAEMA) under a CO_2_ atmosphere in water. While DEAEMA was readily protonated at room temperature via CO_2_ bubbling, it underwent rapid deprotonation upon increasing the reaction temperature to 90 °C. This ease of deprotonation in bicarbonate salts derived from tertiary amines represents a significant advantage in applications requiring rapid reversion to the neutral amine form [27]. In addition to their favorable switching kinetics, tertiary amines benefit from commercial availability and relatively low cost. Bulky primary and secondary amines are also capable of CO_2_-switching behavior through bicarbonate salt formation, with steric hindrance playing a critical role in preventing carbamate formation. The presence of a single bulky alkyl substituent, such as isopropyl, on the nitrogen atom is typically sufficient to inhibit carbamate formation, allowing for rapid switching. Interestingly, these bulky secondary amines often exhibit faster switching dynamics than tertiary amines; however, the incorporation of two bulky groups, as in the case of diisopropylamine, diminishes this kinetic advantage. Moreover, secondary and primary amines offer an additional benefit of enhanced biodegradability compared to their tertiary counterparts [72].

#### 2.2.4. Guanidines Groups

Guanidines represent the most basic class of CO_2_-switchable functional groups, and consequently, the regeneration of their neutral form from the corresponding bicarbonate salts necessitates greater energy input, typically involving higher temperatures and extended heating durations [68]. The incorporation of aromatic substituents can attenuate the inherent basicity of guanidines. Notably, guanidines possessing N–H bonds may form carbamates either exclusively or concurrently with bicarbonate salts upon exposure to CO_2_ [27]. Guanidine–alcohol mixtures have demonstrated CO_2_ sensitivity, functioning as switchable solvents; these mixtures transition into ionic liquids under a CO_2_ atmosphere and revert to their neutral state upon heating in a nitrogen environment [73]. This reversible behavior has inspired the design of guanidine-based CO_2_-responsive polymers. Although the higher temperatures required to revert guanidinium bicarbonate or carbamate salts to their neutral counterparts may pose limitations in certain systems, this thermal stability can be advantageous in applications necessitating elevated operational temperatures [32].

The choice of these functional groups amines, amidines, imidazoles, and guanidines derives from their robust and reversible interaction with CO_2_ under mild conditions. These groups facilitate dynamic alterations in the solubility, conformation, or assembly behavior of polymers upon exposure to CO_2_. Their efficacy, along with synthetic accessibility and compatibility with polymerization methods, positions them as optimal candidates for the development of responsive materials in diverse applications [30,31].

## 3. Synthesis Methods of CO_2_-Responsive Polymers

CO_2_-responsive polymers can be synthesized through a range of radical polymerization techniques, which are generally classified into two categories: conventional free radical polymerization (FRP) and controlled/living radical polymerization methods. While FRP is straightforward and widely used for large-scale production, it offers limited control over molecular weight and architecture. In contrast, controlled radical techniques such as atom transfer radical polymerization (ATRP), reversible addition-fragmentation chain transfer (RAFT), and nitroxide-mediated polymerization (NMP) enable precise control over polymer structure, block formation, and functional group placement. The following sections discuss these techniques with representative examples relevant to CO_2_-responsive applications [55,74].

### 3.1. Free Radical Polymerization (FRP)

Free radical polymerization (FRP) is a significant commercial polymerization process including three primary stages: initiation, propagation, and termination. Free radical polymerization (FRP) is extensively used in diverse polymerization methods, such as bulk, solution, and emulsion polymerization. The flexibility, simplicity, and compatibility of FRP with a wide array of monomers make it a favored technique for manufacturing CO_2_-responsive polymers with customizable characteristics [74,75,76,77]. Early research on CO_2_-switchable polymer systems deploying FRP primarily concentrated on emulsion polymerization for the synthesis of latexes, incorporating CO_2_ responsiveness via surface-active moieties. Fowler et al. (2011) [78] developed CO_2_-switchable polystyrene (PS) and PMMA latexes stabilized by cationic surfactants like N′-hexadecyl-N,N-dimethylacetamidinium bicarbonate. The latexes demonstrated reversible aggregation and redispersion through the alternating bubbling of CO_2_ and N_2_, influenced by the protonation state of the switchable surfactant molecules. Similarly, Mihara and colleagues [79] developed redispersible latexes using imidazole-functionalized initiators capable of reversible protonation when exposed to CO_2_, resulting in electrostatic repulsion strong enough to stabilize latex particles without the need for additional surfactants. Nonetheless, even with their reversible behavior, these systems frequently faced limitations due to low solid content (<7 wt%), incomplete protonation at extreme temperatures, and the hydrolysis of functional groups.

**Figure 2:**
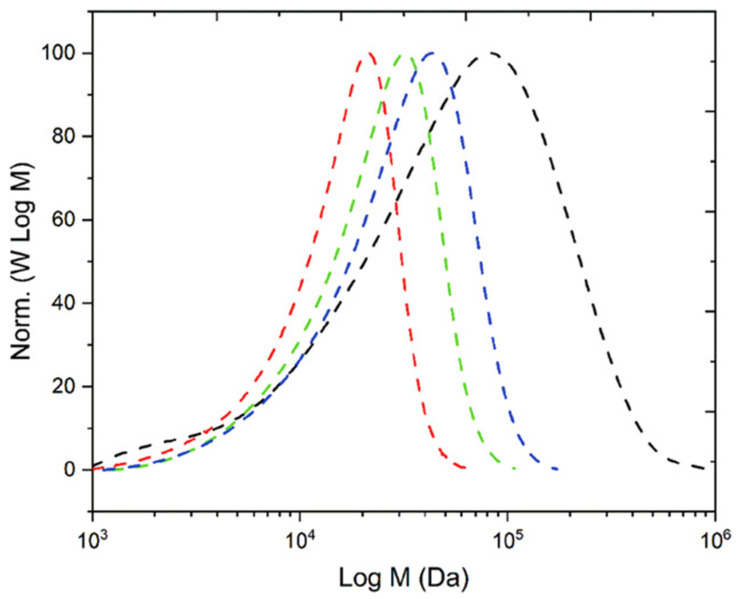
Molecular weight distribution (MWD) profiles of PDMAPAm synthesized via RAFT (CDTPA:AIBN = 3:1 (---), 1.2:1 (---), 0.7:1 (---) and free radical polymerization (---) after 24 h [74].

A further advancement was achieved through the integration of tertiary amine-based comonomers, including 2-(diethylamino)ethyl methacrylate (DEAEMA) and N,N-dimethylaminoethyl methacrylate (DMAEMA), into emulsion formulations. The incorporation of these monomers led to notable enhancements in CO_2_ sensitivity and particle stability. For instance, DEAEMA was utilized in the fabrication of CO_2_-switchable Pickering emulsions and reactive latexes, resulting in materials that could be easily dispersed or aggregated by altering the gas environment. Researchers reported that latexes prepared with VA-044 as the initiator fully protonated under CO_2_ conditions achieved solid contents of over 20% without the need for any comonomer or surfactant, highlighting a significant advancement in the formulation of redispersible latexes [61,76,80,81]. While these foundational studies established FRP as a reliable tool for CO_2_-responsive latex fabrication, recent research has broadened its application scope far beyond emulsions. A prominent example is the work by Pashayev et al. (2024) [74], who synthesized the homopolymer poly(N-[3-(dimethylamino)propyl]acrylamide) (PDMAPAm) via conventional FRP. The monomer, DMAPAm, contains both secondary and tertiary amine functionalities, which undergo reversible protonation and deprotonation upon CO_2_–N_2_ exposure, enabling switchable hydrophilic–hydrophobic behavior. Despite a relatively high dispersity (Đ ≈ 3.8) compared to RAFT-derived analogs, the FRP-based polymer retained effective CO_2_-responsiveness. This highlights the strength of the functional monomer design in facilitating stimuli-responsive behavior, even when produced via less controlled radical methods. Figure 2 illustrates that the molecular weight distribution of the FRP-synthesized PDMAPAm is broader compared to the RAFT-synthesized versions. However, the functional performance regarding CO_2_-triggered switching remains intact, highlighting the potential of FRP for scalable and cost-effective production of CO_2_-responsive polymers.

**Figure 3:**
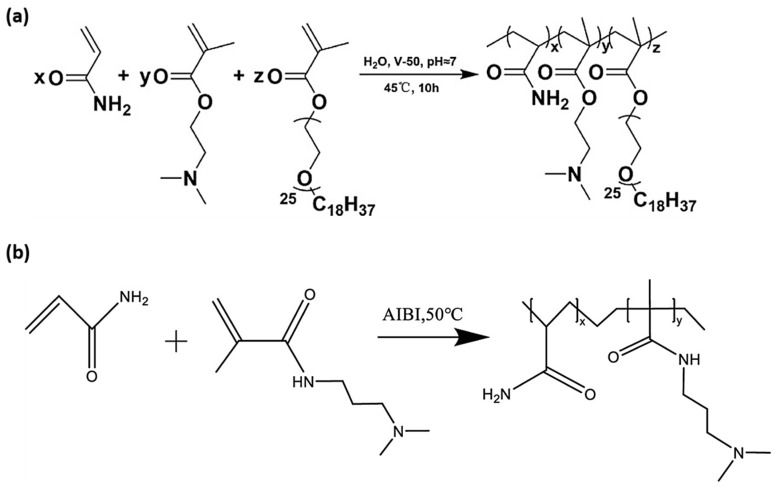
(**a**) Synthesis of CO_2_-responsive polymer (**a**) via aqueous FRP of DMAEMA, AM, and polyether methacrylates [75] (**b**) by using gel using AM, DMAPMA, and PEI via aqueous FRP [82].

In another significant study, Huang et al. (2024) [75] developed CO_2_-responsive polymers by applying aqueous free radical copolymerization of DMAEMA and polyether-modified methacrylates with acrylamide at 45 °C, as illustrated in Figure 3a. The resulting polymers featured tertiary amine groups and hydrophobic polyether side chains, which endowed them with gas-switchable solubility and dual responsiveness to both pH and CO_2_, making them attractive for smart hydrogel and coating applications. Likewise, Wu et al. (2023) [82] developed a CO_2_-responsive intelligent polymer sealant by using aqueous FRP of acrylamide and DMAPMA, which was cross-linked with polyethyleneimine (PEI) to improve its functionality (Figure 3b). The resulting polymer gel exhibited reversible swelling behavior and strong pH and CO_2_ responsiveness.

**Figure 4:**
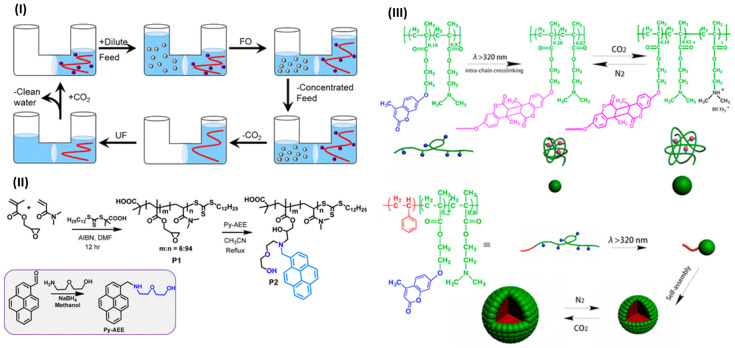
(**I**–**III**) Representative examples of CO_2_-responsive polymer systems synthesized via free radical polymerization (FRP). (**I**) Forward osmosis–ultrafiltration (FO–UF) process using CO_2_-switchable nitrogen-rich polyamines as draw solutes [84]. (**II**) Synthesis of pyrene-functionalized polymeric probe P2 for CO_2_–pH-tunable fluorescence [85]. (**III**) Preparation and CO_2_–N_2_-responsive behavior of single-chain nanoparticles and micellar aggregates from DMAEMA-based copolymers [86].

In the realm of nanostructured polymer systems, Yu et al. (2019) [83] revealed an innovative enzyme-assisted, photo-initiated FRP (photo-PISA) process carried out under open-air conditions. Cross-linked CO_2_-responsive polymer vesicles were synthesized through the copolymerization of HPMA and DMAEMA using a photoinitiator and an oxygen-scavenging enzyme system. The addition of the asymmetric cross-linker allyl methacrylate (AMA) provided mechanical stability, whereas DMAEMA units granted the vesicles CO_2_-switchable expansion behavior. Zhao et al. (2017) [48] investigated the new class of FRP-derived polymeric surfactants that include DEAEMA, demonstrating the ability to alter emulsification properties in oil–water systems based on the CO_2_–N_2_ gas environment. This study broadened the use of FRP-based CO_2_-responsive polymers in dynamic emulsion stabilization, an important field in materials chemistry. In another significant development, Ellis et al. (2019) [84] used FRP to synthesize nitrogen-rich polyamine draw solutes for forward osmosis, allowing for the modulation of osmotic pressure and CO_2_-responsiveness through the protonation of amine groups (Figure 4I). Furthermore, Gupta and Lee (2017) [85] developed a pyrene-functionalized polymeric probe through FRP for the fluorescence-based detection of nerve agent mimics (Figure 4II). The fluorescence of the material can be reversibly activated and deactivated through CO_2_–N_2_ purging, which is linked to the protonation state of amine groups affecting the photoinduced electron transfer (PET) pathway. Lastly, Fan et al. (2017) [86] developed single-chain CO_2_-responsive polymer nanoparticles (SCNPs) with free radical polymerization of copolymers, including DMAEMA (Figure 4III). Reversing swelling and shrinking characteristics of the nanoparticles allowed for gas-tunable control over their size and catalytic activity.

Collectively, these contemporary examples illustrate that FRP is not merely a legacy method for latex production but a versatile and robust tool for constructing CO_2_-responsive polymers in various advanced formats. Despite its lack of molecular-level control compared to living radical polymerizations like RAFT or ATRP, FRP remains highly effective in producing functionally responsive materials, especially when precision in molecular weight is not a limiting factor for application.

### 3.2. Controlled Radical Polymerization Techniques

Reversible deactivation radical polymerization (RDRP), or controlled/living radical polymerization, has transformed polymer synthesis by permitting precise regulation of molecular weight, dispersity, and polymer architecture. In contrast to conventional living polymerizations, including anionic or cationic techniques, reversible-deactivation radical polymerization (RDRP) functions under more accessible and practical conditions [87]. In FRP, the lifespan of the developing chains is very rapid (under 1 s), resulting in all polymer chains becoming “dead” after the reaction. Consequently, the polymerization is non-living, and block copolymers cannot be produced by this process [88]. Anionic polymerization, as developed by Szwarc [89], reflects a true living system in which chain transfer and termination reactions are effectively eliminated. This can only be accomplished by removing all moisture and/or oxygen, requiring high vacuum conditions and very low temperatures (approximately −80 °C) [90]. By utilizing RDRP, or living radical polymerization, various monomers can be polymerized under milder conditions commonly used in FRP, while achieving a substantial level of control over the polymer structures. Additionally, RDRP exhibits lower sensitivity to water compared to anionic polymerization, which represents a significant advantage, as numerous industrial polymerizations occur in aqueous environments, such as emulsion polymerizations for latex production. In RDRP, the minimal occurrence of irreversible termination reactions allows for chains to remain active for extended durations, ideally throughout the entire polymerization process. This enables the incorporation of additional blocks, facilitating the preparation of block copolymers [89].

Subsequent sections will demonstrate that RDRP has emerged as the preferred polymerization technique for the synthesis of CO_2_-switchable polymers characterized by complex microstructures or mesostructures, such as di- and triblock copolymers and nanostructured particles. FRP is a more straightforward and cost-effective option compared to RDRP, particularly when precise control over molecular weight distribution and complex molecular architectures is not essential.

**Figure 5:**
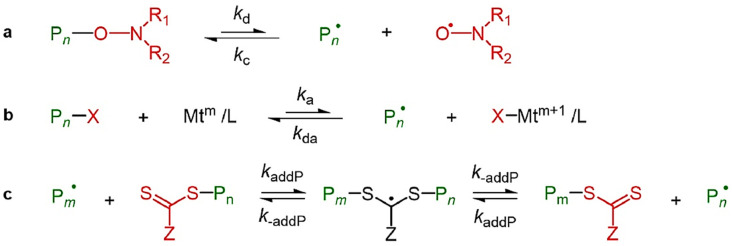
Overview of simplified activation and deactivation mechanisms in reversible deactivation radical polymerization (RDRP). (**a**) Polymerization mediated by nitroxide radicals (NMP); (**b**) atom transfer radical polymerization (ATRP); (**c**) reversible addition-fragmentation chain transfer (RAFT) polymerization [87].

The three predominant types of reversible-deactivation radical polymerization (RDRP) are nitroxide-mediated polymerization (NMP), atom transfer radical polymerization (ATRP), and reversible addition-fragmentation transfer (RAFT) polymerization. Figure 5 provides a clear summary of these mechanisms, illustrating the activation–deactivation equilibria in NMP, ATRP, and RAFT polymerization pathways [87]. The primary distinction between RDRP and FRP lies in the significant reduction of termination reactions in RDRP, achieved through the incorporation of a control agent that facilitates the reversible deactivation of propagating chains. In NMP, nitroxide is introduced to the system to deactivate propagating radicals and reduce mutual termination. ATRP employs a transition metal catalyst, whereas RAFT utilizes a chain transfer agent to decrease the occurrence of termination reactions. NMP and ATRP utilize reversible termination, whereas RAFT employs reversible chain transfer [91]. Each of these controlling mechanisms can yield a polymer characterized by low dispersity (Đ < 1.5). Numerous reviews of RDRP across various polymerization systems, including dispersed systems, have been published [87,91,92,93]. The application of RDRP techniques in the production of switchable materials is emerging as a significant research focus. This paper highlights the synthesis of CO_2_-switchable polymers through reversible-deactivation radical polymerization (RDRP) in the subsequent sections.

#### 3.2.1. Atomic Transfer Radical Polymerization (ATRP)

**Figure 6:**
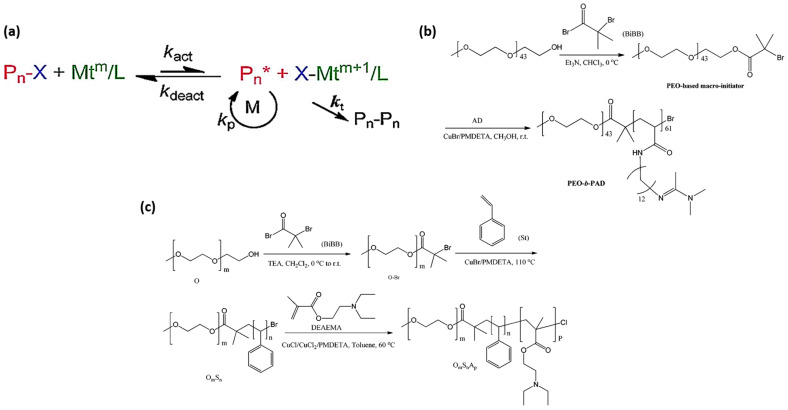
(**a**–**c**) ATRP-based synthetic approaches for constructing CO_2_-responsive polymer architectures. (**a**) Illustration of the ATRP equilibrium mechanism [95]. (**b**) Schematic synthesis pathway of the diblock copolymer PEO-b-PAD [96]. (**c**) Stepwise ATRP strategy for preparing the triblock copolymer PEO-b-PS-b-PDEAEMA [98].

Atom transfer radical polymerization (ATRP) is a prevalent method for the controlled radical polymerization of CO_2_-responsive polymers. In ATRP, there is an equilibrium between propagating radicals and dormant species [94]. The dormant species (Pn–X) are common and intermittently interact with transition metal complexes. Transition metal complexes function as activators in their lower oxidation state (Mtm/L), engaging with halide-terminated “dormant” chains to produce propagating radicals (Pn˙) and transition metal complexes in a higher oxidation state. In elevated oxidation states, transition metal complexes coordinate with halide ligands (X–Mtm+1/L) and function as deactivators. The deactivator interacts with propagating radicals to produce dormant species and the activator (Figure 6a). Consequently, the concentration of propagating radicals remains low in comparison to FRP, leading to a reduction in termination reactions. In certain applications, it may be necessary to remove the transition metal catalyst and ligand. This is clear for homogeneous systems; however, it can cause challenges in heterogeneous systems [95].

The versatile nature of ATRP has allowed for the development of a wide spectrum of CO_2_-switchable polymer systems. Classical copper-mediated ATRP has been utilized to synthesize diverse diblock, triblock, star-like, and surface-grafted CO_2_-responsive polymers [96]. Poly(ethylene oxide)-block-poly((N-amidino)dodecyl acrylamide) (PEO-b-PAD) was synthesized using ATRP at room temperature using CuBr/PMDETA, providing a well-defined diblock structure containing CO_2_-switchable amidine moieties. Figure 6b illustrates the synthesis route for the preparation of PEO-b-PAD. The reaction was conducted in anhydrous methanol for 12 h, deploying pentamethyldiethylenetriamine (PMDETA) as the ligand and copper bromide (CuBr) as the catalyst [96]. Likewise, triblock copolymers like PEO-b-PS-b-PDEAEMA have been synthesized using sequential ATRP, using the tertiary amine functionality of DEAEMA for CO_2_-responsiveness. The PEO macroinitiator was prepared by conducting the ATRP of mono-methoxyl poly(ethylene oxide) in dichloromethane (CH_2_Cl_2_) with triethylamine (TEA) at ambient temperature for 24 h, using 2-bromo-isobutyryl bromide (BiBB) as the initiator. The macroinitiator (O–Br) was used in the ATRP of styrene in bulk using copper bromide (CuBr) as the catalyst and PMDETA as the ligand (Figure 6c). The reaction persisted for 16 h at 110 °C to yield the OS macroinitiator for the third stage. The last phase was the ATRP of DEAEMA conducted in toluene with an OS macroinitiator, a combination of copper chlorides (CuCl and CuCl_2_) as the catalyst, and the PMDETA ligand at 60 °C for 24 h. The PMDETA ligand was used in the production of a PDMAEMA-b-PEO-b-PDMAEMA triblock copolymer, functioning as a CO_2_-switchable nanocomposite via ATRP using a Br–PEO–Br initiator [97].

Likewise, Xu et al. (2013) [99] synthesized a fluorophore–polymer conjugate, PBI–PDMAEMA, which consists of two CO_2_-switchable PDMAEMA arms and a central perylene-3,4,9,10-tetracarboxylic acid bisimide (PBI) fluorophore. This was achieved through the ATRP of DMAEMA using N,N′-bis{2-[2-[(2-bromo-2-methylpropanoyl)oxy]ethoxy]ethyl}perylene-3,4,9,10-tetracarboxylic acid bisimide initiators. Following the synthesis of the PBI–Br macroinitiator (ATRP macroinitiator), it was applied in the ATRP of DMAEMA with CuBr as the catalyst, PMDETA as the ligand, and THF as the solvent.

**Figure 7:**
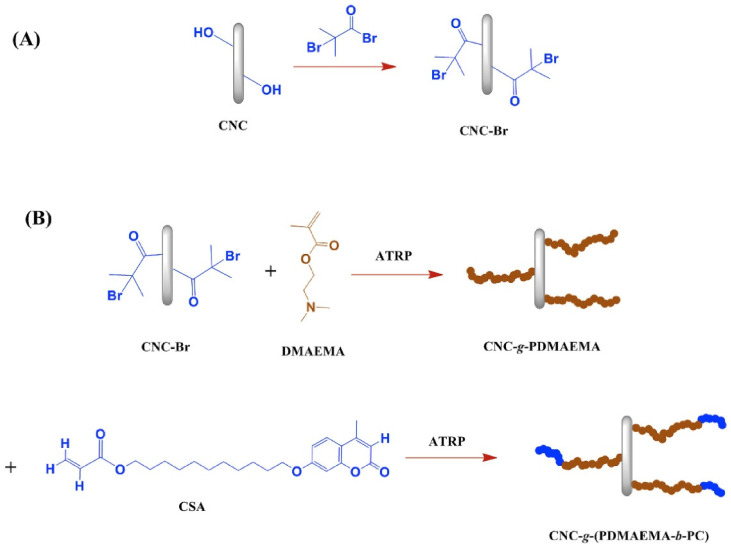
Synthesis of (**A**) CNC-Br and (**B**) CNC-g-(PDMAEMA-b-PC) [52].

In a recent study, Rezvani et al. (2019) [52] synthesized CO_2_-responsive polymers using the ATRP method. The process commenced with the extraction of cellulose nanocrystals (CNC) from microcrystalline cellulose (MCC) via acid hydrolysis. The CNCs were subsequently functionalized through the grafting of an ATRP initiator, α-Bromoisobutyryl bromide (BiBB), leading to the formation of CNC-Br. This step offered the essential spots for starting polymerization. The subsequent phase entailed the synthesis of copolymers through the grafting of poly(2-dimethylaminoethyl methacrylate) (PDMAEMA) and coumarin derivatives onto the CNCs. The reaction took place in tetrahydrofuran (THF), with DMAEMA and a coumarin derivative (CSA) added as monomers, while a catalyst system consisting of CuBr and PMDETA was used to initiate the polymerization (Figure 7). The polymerization took place at 70 °C for 24 h, leading to the production of both free copolymers and CNC-grafted copolymers.

**Figure 8:**
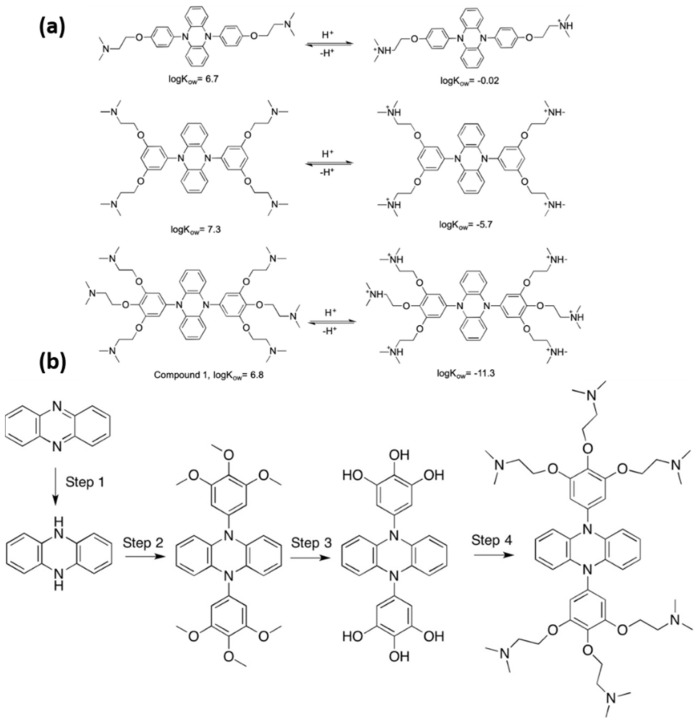
(**a**) Molecular structures and predicted log Kow values for three potential CO_2_−switchable photocatalysts with varying numbers of tertiary amine groups in their neutral and protonated forms. (**b**) Synthesis of the CO_2_−switchable photoinitiated catalyst (compound 1): 2,2′,2″,2‴,2⁗,2′′′′′-[Phenazine-5,10−diyl bis [(benzene-5,1,2,3-tetrayl)tris(oxy)]] hexakis (N,N-dimethylethan−1-amine) [100].

A recent innovation is the development of organocatalyzed ATRP (O-ATRP), which removes the necessity for metal catalysts. Su et al. (2019) [100] introduced an innovative method for synthesizing both hydrophobic and hydrophilic polymers through organocatalyzed atom transfer radical polymerization (O-ATRP). This approach uses a CO_2_-switchable photocatalyst capable of reversible transitions between hydrophobic and hydrophilic states, contingent on the presence or absence of CO_2_. This catalyst’s primary characteristic is its ability to be flipped by the addition or removal of CO_2_, allowing for reuse in future polymerization processes. The photocatalyst, functionalized with tertiary amine groups, exhibits hydrophobic properties in its neutral state (CO_2_-absent) and transitions to a hydrophilic state upon protonation in the presence of CO_2_. The polymerization can be conducted in either organic or aqueous media, depending on the state of the catalyst (Figure 8a). The development of the CO_2_-switchable photocatalyst (compound 1) included the functionalization of diphenyldihydrophenazine with tertiary amines to facilitate CO_2_ responsiveness. The catalyst demonstrates efficacy in hydrophobic polymerizations using solvents such as toluene, as well as in hydrophilic polymerizations conducted in carbonated water. The authors demonstrated that the catalyst could be effectively recycled and reused across multiple cycles, with minimal residual catalyst present in the final polymer (less than 15 ppb).

The O-ATRP process was conducted using styrene (St) in toluene and hydroxyethyl methacrylate (HEMA) in carbonated water, demonstrating effective control over polymerization and resulting in narrow molecular weight distributions. The authors investigated the recycling process, in which the catalyst was extracted from the polymerization mixture relying on CO_2_-saturated water, and following the removal of CO_2_, it was employed again in later polymerizations. Figure 8b illustrates the synthesis of the CO_2_-switchable photocatalyst (compound 1), which highlights the recycling process of the catalyst following polymerization. This novel method offers a more sustainable approach to ATRP by enabling the reuse of the catalyst and reducing environmental impact, especially through the utilization of CO_2_ as a trigger for transitioning between hydrophilic and hydrophobic states [100].

#### 3.2.2. Reversible Addition-Fragmentation Chain Transfer (RAFT) Polymerization

**Figure 9:**
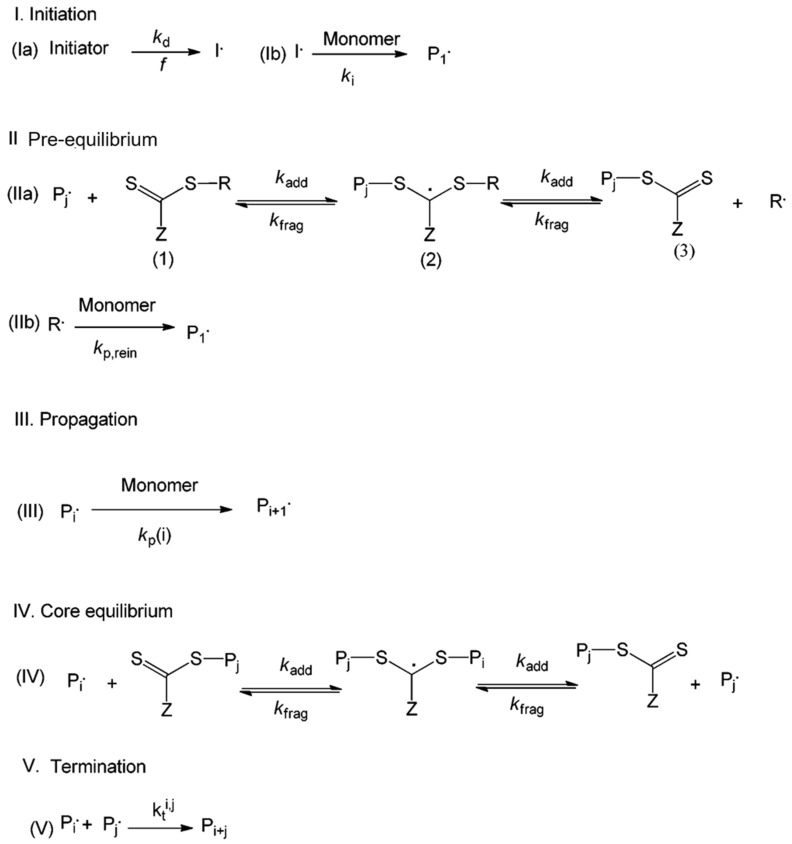
Schematic depiction of the RAFT polymerization mechanism [101]. (**Ia**) Initiation; (**Ib**) formation of propagating radicals. (**IIa**,**b**) Pre-equilibrium step forming macro-RAFT agents. (**III**) Chain propagation. (**IV**) Main RAFT equilibrium involves chain transfer between active and dormant species. (**V**) Termination [101].

RAFT polymerization is a highly versatile and widely used technique in the field of reversible deactivation radical polymerization (RDRP) methods, particularly effective for the synthesis of CO_2_-responsive polymers with exact structural control. RAFT, established in the late 1990s, uses thiocarbonylthio-based chain transfer agents (CTAs) to promote a degenerative chain transfer process involving dormant and active chains. This promotes consistent chain growth, even at higher conversions, resulting in polymers characterized by narrow molecular weight distributions and regulated architectures [101]. Figure 9 illustrates a standard kinetic scheme for RAFT polymerization. Reaction I serves as the initiation step in RAFT, analogous to FRP. During the pre-equilibrium step (reaction II), the transfer of the RAFT agent to a propagating chain results in the formation of a macroRAFT agent. Reaction IV illustrates the principal RAFT equilibrium, in which the RAFT agent is exchanged among advancing polymer chains. Propagation (reaction III) and termination (reaction V) occur in a way comparable to the FRP mechanism. RAFT accommodates a broader range of monomers compared to NMP or ATRP, making it a commonly employed technique for synthesizing well-defined (co)polymers characterized by narrow molecular weight distributions and specific end functionalities.

The RAFT agent can introduce color to the product, and residual odor can be a concern, which requires the post-reaction removal of the RAFT agent from the polymer chain ends [101].

Lin et al. (2016) [102] explained a straightforward grafting method in their paper that integrates RAFT polymerization with post-polymerization modification for the synthesis of CO_2_-responsive graft copolymers. A linear copolymer of poly(N,N-dimethylacrylamide-co-pentafluorophenyl acrylate), referred to as P(DMA-co-PFPA), was synthesized using RAFT polymerization. The backbone featured reactive pentafluorophenyl ester groups, providing the subsequent grafting of amine-terminated poly(2-(diethylamino)ethyl methacrylate) (PDEAEMA), a recognized CO_2_-responsive polymer, along with optional poly(methyl methacrylate) (PMMA) side chains. The homo-graft (PDMA-g-PDEAEMA, P4) and hetero-graft (PDMA-g-(PDEAEMA; PMMA), P5) copolymers demonstrated self-assembly into vesicular structures in aqueous media. When CO_2_ was bubbled through, the vesicles expanded as a result of the protonation of tertiary amine groups on the PDEAEMA side chains. P4 demonstrated reversible swelling and shrinking when subjected to alternating purging with CO_2_ and Ar, whereas P5 exhibited irreversible aggregation attributed to the presence of hydrophobic PMMA segments.

In another study by Guo et al. (2022) [103], a new approach to switchable RAFT polymerization was developed to fabricate CO_2_-responsive gradient copolymers, allowing for precise control over the sequence distribution of the monomers. This method involved a proton-switchable RAFT agent that could selectively polymerize more-activated monomers (MAMs) or less-activated monomers (LAMs) by switching between their active states through an acid–base treatment. Using this technique, the researchers synthesized a triblock copolymer, PBzMA-b-P(DEAEMA-grad-NVP)-b-PNVP, where the middle block showed a gradient from hydrophobic DEAEMA to hydrophilic NVP. This gradient structure led to the formation of compartmentalized micelles with CO_2_-responsive properties. These copolymers exhibited unique morphologies compared to typical block copolymers, offering the potential for tunable self-assembly and morphological changes when exposed to CO_2_.

**Figure 10:**
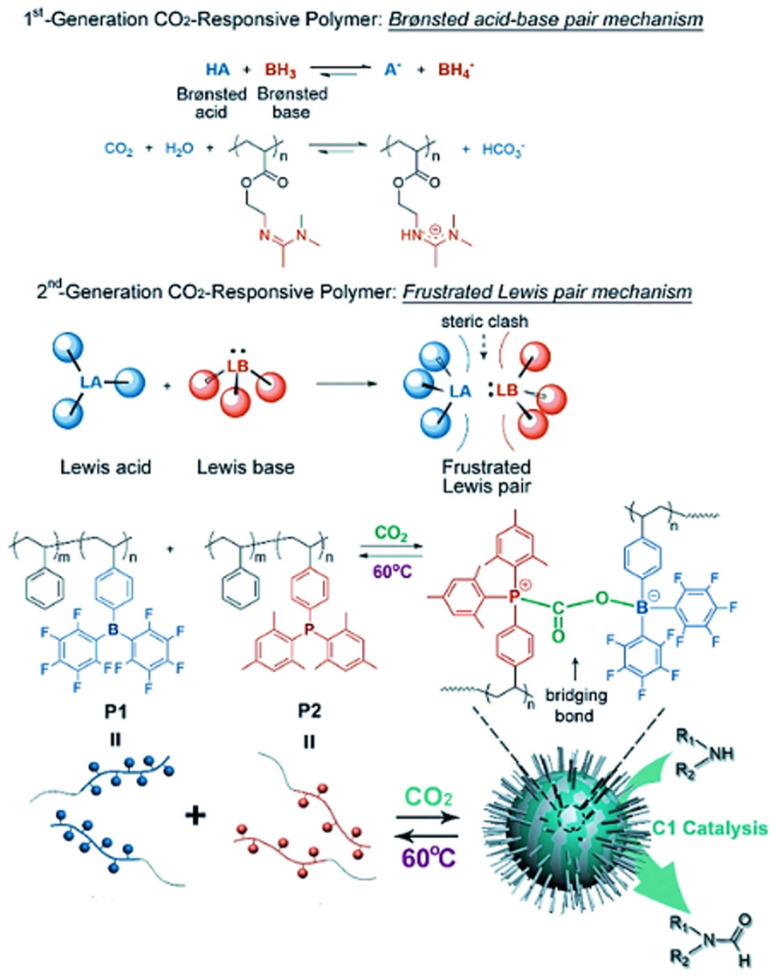
(**1st**) Working principle of first-generation CO_2_-responsive polymers based on Brønsted acid–base pairs, with a typical example of amidine-containing polymers. (**2nd**) Frustrated Lewis pair (FLP) mechanism and the design of FLP-containing block copolymers (P1 and P2) as second-generation CO_2_-responsive systems for CO_2_-activated micellization and as recyclable nanocatalysts for CO_2_ catalytic conversion [104].

In a similar vein, Chen et al. (2018) [104] presented a second-generation CO_2_-responsive system based on frustrated Lewis pair (FLP) chemistry, also synthesized via RAFT polymerization. In their system, two complementary block copolymers were created: one with bulky borane groups (Lewis acid) and the other with phosphine groups (Lewis base). When CO_2_ was bubbled through the mixture, these components formed cross-linked micelles via dative bonding, with CO_2_ acting as a bridge between the two blocks. These FLP-based micelles demonstrated an impressively fast CO_2_-responsiveness (less than 20 s), as well as thermal reversibility and recyclability. Moreover, the micelles were used as nanocatalysts in the conversion of C1 feedstocks, opening up new possibilities in sustainable chemistry. The process of self-assembly and catalysis with these FLP-based micelles is depicted in Figure 10.

Amphiphilic block copolymers composed of poly(methacrylic acid) (PMAA) and poly(2-dimethylaminoethyl methacrylate) (PDMAEMA) serve as emulsifiers in the RAFT-mediated emulsion polymerization of PMMA latex particles. Through the modification of PDMAEMA block length and pH during polymerization, latex particles with surfaces enriched in either PDMAEMA or PMAA were synthesized in another study. The particles demonstrated bidirectional responsiveness to CO_2_; those with PDMAEMA surfaces precipitated at elevated pH levels and redispersed on CO_2_ exposure, but PMAA-surfaced particles exhibited the inverse behavior. This dual responsiveness helped reversible transitions between dispersion and precipitation, presenting a novel approach for intelligent colloidal systems [60].

Table 1 presents a summary of different CO_2_-responsive polymers synthesized through RAFT polymerization, a controlled and living radical polymerization method [105]. These polymers often include functional groups like tertiary amines, amidines, or guanidines that interact with CO_2_ via reversible chemical processes. RAFT allows for the accurate regulation of molecular weight, block composition, and end-group functionality, rendering it particularly effective for the synthesis of block copolymers, micelles, and nanostructures that experience CO_2_-induced phase transitions or self-assembly [103].

#### 3.2.3. Nitroxide-Mediated Polymerization (NMP)

Nitroxide-mediated polymerization (NMP) represents one of the earliest and strongest-established techniques among controlled radical polymerization (CRP) methods. NMP relies on a stable nitroxide radical, commonly TEMPO or analogous compounds, to reversibly deactivate growing polymer chains, which promotes polymerization with minimal termination. NMP offers a significant advantage over traditional free radical polymerization (FRP) by enabling precise control over molecular weight and producing narrow molecular weight distributions [115]. In the 1980s, Solomon et al. identified that radicals produced during free radical polymerization can be captured by nitroxides, which leads to the formation of controlled and living low molecular weight polymers [116]. In 1993, Georges and colleagues [117] applied 2,2,6,6-tetramethylpiperidinyl-N-oxyl (TEMPO) as a nitroxide in the living radical polymerization of styrene, leading to polystyrene with a narrow molecular weight distribution. This research established the basis for nitroxide-mediated polymerization. In NMP, an alkoxyamine encounters homolytic decomposition, resulting in the formation of nitroxide and initiator radicals [118].

Nitroxides are stable radicals that typically do not self-terminate, but they can react with growing radicals to deactivate them. The activation of a nitroxide-terminated polymer chain occurs roughly every 10^2^ to 10^3^ s, while deactivation happens quickly, around ∼10^−3^ s. During the brief period between activation and deactivation, a small number of monomers (usually 1 to 5) are added to the growing chains, resulting in polymers with a narrow molecular weight distribution (MWD) [90]. While nitroxides do not participate in irreversible termination reactions, propagating radicals may still terminate through mutual interactions. As conversion progresses, some irreversible termination occurs, increasing the ratio of nitroxide radicals to propagating radicals. Alkoxyamines typically require activation at elevated temperatures (>90 °C). Therefore, using nitroxide-mediated polymerization (NMP) to synthesize CO_2_-switchable polymers in a CO_2_ atmosphere is not practical, as CO_2_-switchable monomers would be protonated in that environment. High pressure would be needed to ensure sufficient CO_2_ dissolution. Alternatively, CO_2_-switchable groups can be protonated with strong acids, such as HCl, but this may cause the polymers to lose their CO_2_-switchable properties unless the acid is neutralized with a base and the residual salts are washed away.

**Figure 11:**
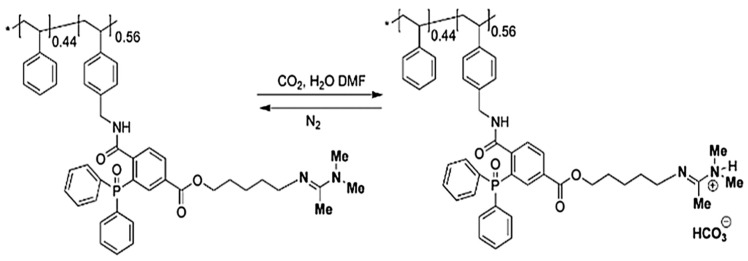
Reversible protonation and deprotonation of p(“amidine”MS) −co−PS in DMF with 0.5% H_2_O following alternating CO_2_ and N_2_ purging [119].

Well-defined CO_2_-switchable polymers can be synthesized via NMP in a nitrogen atmosphere. For example, Zhou et al.(2009) [119] synthesized poly(p-chloromethylstyrene)-co-polystyrene (PCMS-co-PS) with low molar dispersity using NMP. They then modified this polymer to poly(p-azidomethylstyrene)-co-polystyrene (PAMS-co-PS) and introduced amidine groups through Staudinger ligation. This polymer demonstrated a neutral–charged–neutral transition in DMF with 0.5% H_2_O by alternately introducing CO_2_ and N_2_, which was confirmed by noticeable changes in the polymer’s conductivity (Figure 11).

Recently, Glasing et al. (2017) [120] used a grafting methodology to alter cellulose nanocrystals (CNC) with CO_2_-responsive polymers using nitroxide-mediated polymerization (NMP). This method involved the functionalization of the CNC surface with SG1-capped poly(2-dimethylaminoethyl methacrylate) (PDEAEMA) and poly(N-3-(dimethylamino)propyl methacrylamide) (PDMAPMAm) macroalkoxyamines. The synthesis of these CO_2_-responsive polymers was conducted in bulk via NMP, facilitating precise control over molecular weight, dispersity, and end-group fidelity. The grafting method enabled a comprehensive analysis of the preformed functional polymers before their attachment to the CNC surface, the initial step in the synthesis of PDEAEMA and PDMAPMAm, using NHS-BlocBuilder and 10 mol% styrene as a co-monomer in bulk at a temperature of 80 °C. This step indicates the polymerization of the monomers PDEAEMA and PDMAPMAm using the SG1-capped nitroxide as a radical initiator, facilitating well-controlled polymerization. Subsequently, CNC was modified with Glycidyl methacrylate (GMA), which is essential for introducing reactive sites on the CNC surface, thereby facilitating the subsequent grafting of CO_2_-responsive polymers. The modification of the CNC surface with GMA is essential for the subsequent Surface-Initiated NMP (SI-NMP) process, in which the functionalized CNC acts as a macro-initiator for the growth of CO_2_-responsive polymer chains. The CO_2_-responsive graft copolymers demonstrated reversible alterations in surface properties when exposed to CO_2_, shifting between hydrophobic and hydrophilic states. The ability to switch behavior presents opportunities for applications in nanocomposites, drug delivery systems, and wastewater treatment, where the modulation of surface properties is advantageous [120].

In a further investigation, surface-initiated nitroxide-mediated polymerization (SI-NMP) was chosen to graft CO_2_-responsive polymers onto cellulose nanocrystals (CNCs). CNCs were initially functionalized using an SG1-based alkoxyamine (CNC-BB), which acted as a macroinitiator. The functionalized CNCs underwent polymerization with 2-dimethylaminoethyl methacrylate (DMAEMA), diethylaminoethyl methacrylate (DEAEMA), and 3-(dimethylamino)propyl methacrylamide (PDMAPMAm) in a nitrogen atmosphere at 85 °C, utilizing styrene as a co-monomer to enhance polymerization control. The grafted CO_2_-responsive polymers demonstrated reversible transitions between hydrophilic and hydrophobic states when exposed to CO_2_. The full process starts with the synthesis of CNC–macroalkoxyamine (CNC–BB) and the subsequent grafting polymerization of DMAEMA, DEAEMA, and PDMAPMAm onto the CNC surface via SI-NMP. This method allows for the precise control of polymer grafting, enabling the creation of CO_2_-responsive CNC-based materials [53].

Table 2 provides a comparative analysis of the primary polymerization methods used for the synthesis of CO_2_-responsive polymers. Conventional free radical polymerization (FRP) continues to be popular because of its straightforward nature and affordability, particularly for the production of homopolymers or random copolymers. Nonetheless, it provides restricted control over molecular architecture. In contrast, controlled radical polymerization techniques like ATRP, RAFT, and NMP offer precise control over molecular weight, composition, and block architecture, which are essential for customizing CO_2_-responsiveness in self-assembled systems and stimuli-responsive applications. For example, ATRP facilitates the synthesis of precisely defined block copolymers with functional chain ends, whereas RAFT provides remarkable versatility for a wide array of monomers and reaction conditions. Consequently, these controlled methods are more appropriate when reproducibility, responsiveness, and functional complexity are essential in different applications. The comparative table effectively emphasizes their advantages and disadvantages [121,122].

## 4. Applications of CO_2_-Responsive Polymers

The discovery of the potential for designing novel polymeric materials with CO_2_-responsiveness is going to contribute to the growth of innovative applications. This section investigates recent applications and identifies potential opportunities for the development of new materials. Few studies have been published that explore the potential applications of CO_2_-responsive polymers in different fields, which will be thoroughly discussed in this section.

### 4.1. Oil-Water Separation

**Figure 12:**
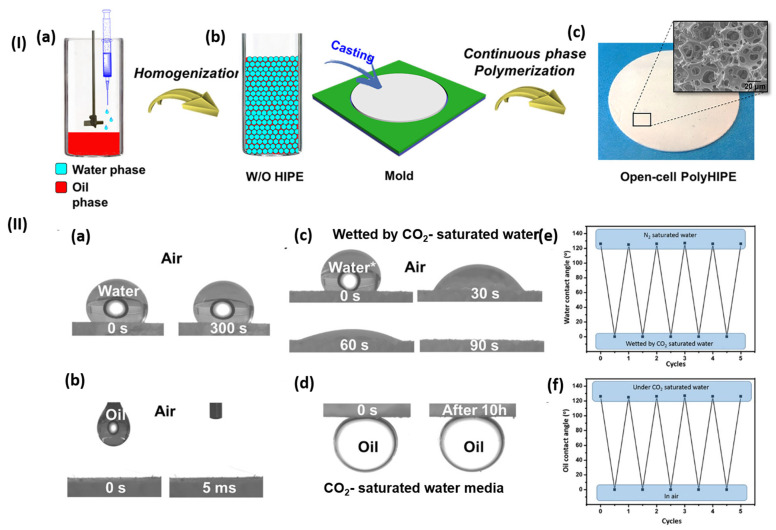
Poly(St-co-DEA)-HIPE membrane: (**I**) synthesis and (**II**) wettability transitions. (**I**) Synthesis: (**a**) water phase added during oil phase homogenization for W/O HIPE preparation; (**b**) HIPF cast into a mold; (**c**) porous polyHIPE membrane formed by polymerizing the continuous phase. (**II**) Wettability measurements: (**a**) natural water contact angle at 0 and 300 s; (**b**) oil contact angle at 0 and 0.5 ms; (**c**) CO_2_-saturated water contact angle at 0, 30, 60, and 90 s; (**d**) oil contact angle under CO_2_-saturated water; (**e**) CO_2_-induced reversible water wettability; (**f**) CO_2_-induced reversible oil wettability [124].

Separation methods constitute a fundamental aspect of numerous chemical industries, typically executed through methods such as extraction, distillation, crystallization, membrane transport processes, and chromatography [123]. CO_2_-responsive polymers hold significant potential for application in advanced oil–water separation systems. Lei et al. (2017) [124] developed highly porous poly(styrene-co-N,N-(diethylamino)ethyl methacrylate) membranes characterized by “open-cell” structures and CO_2_-switchable wettability, applying water-in-oil high internal phase emulsion templates for their preparation (Figure 12I). These innovative membranes can alter their wettability from hydrophobic–superoleophilic to hydrophilic–superoleophobic via straightforward CO_2_ treatment in aqueous systems, providing gravity-driven and controllable oil–water separation. The practical implementation signifies a simple process in their original hydrophobic state, and these membranes permit selective oil penetration while obstructing water. Following CO_2_ treatment, the wettability transitions, allowing for water to permeate while obstructing oil (Figure 12II). This method offers a cost-effective method for the smart separation of large volumes.

**Figure 13:**
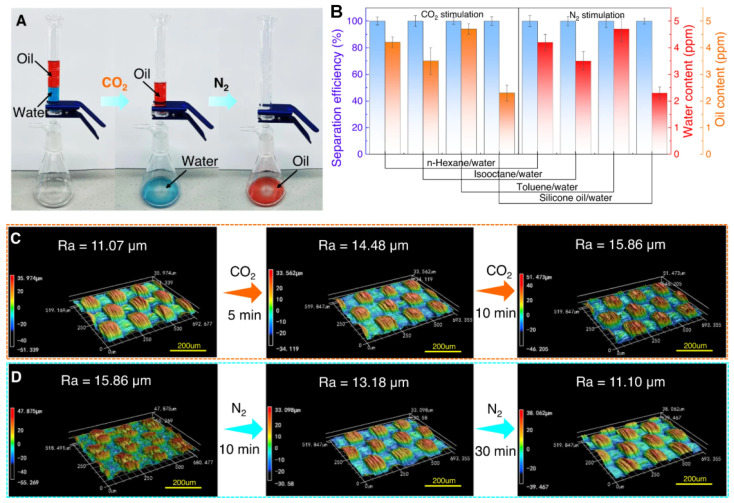
Gas-switchable separation of immiscible oil–water mixtures. (**A**) Separation process un−er CO_2_–N_2_ stimulation at 25 °C. (**B**) Efficiency and oil–water content of PPFM−0.5 (150 μm gap) for four mixtures under CO_2_–N_2_. (**C**,**D**) Surface roughness changes of PPFM-0.5 under CO_2_–N_2_. Error bars show standard deviation from at least three samples [42].

Researchers at Queen’s University Canada have further advanced this technology by developing CO_2_-responsive polymer-grafted cotton using atom transfer radical polymerization (ATRP), demonstrating successful switching between hydrophobic and hydrophilic states when exposed to or removed from carbonated water [125]. Wang et al. (2023) [42] advanced the work of Lei et al. (2017) by developing scalable CO_2_-responsive membranes for the separation of oil and water. The membranes, composed of PMMA-co-PDEAEMA copolymers, demonstrate CO_2_-switchable wettability, shifting between hydrophobic–superoleophilic and superhydrophilic–underwater superoleophobic states in response to CO_2_–N_2_ stimulation. The membranes facilitate the gravity-driven separation of diverse oil–water systems, achieving separation efficiencies exceeding 99.9%. This innovation, observed through a capillary force-driven self-assembling method, provides a cost-effective and scalable solution for efficient and reversible oil–water separation. The membrane exhibits self-cleaning properties and high recyclability, rendering it appropriate for large-scale applications. Figure 13 demonstrates the CO_2_-induced wettability transition, which emphasizes the gas-switchable separation mechanism.

Table 3 presents a summary of CO_2_-responsive polymers applied to oil–water separation applications, outlining the polymer materials, preparation methods, CO_2_-induced wettability alterations, oil–water separation efficacy, and significant findings.

### 4.2. Carbon Capture and Storage

CO_2_-responsive polymers play a significant role in carbon capture and storage (CCS) technologies. Lu et al. [130] developed a more effective polymer, poly(N-heterocyclic carbenes-co-styrene), for CO_2_ capture. The findings indicate that this polymer exhibits enhanced CO_2_ capture rates at relatively low CO_2_ concentrations and demonstrates a more rapid CO_2_ release at elevated temperatures. Recently, Rieger’s group synthesized an acylated polyethyleneimine with amine groups, exhibiting thermal and CO_2_ responsive behaviors for CO_2_ capture and release. The results indicated that the thermal-responsive properties of the polymer facilitate CO_2_ release due to the formation of additional protons during the phase transition (LCST) on heating, which induced the decomposition of ammonium bicarbonate moieties, leading to the easier release of CO_2_ from the acylated polyethyleneimine solution [131].

**Figure 14:**
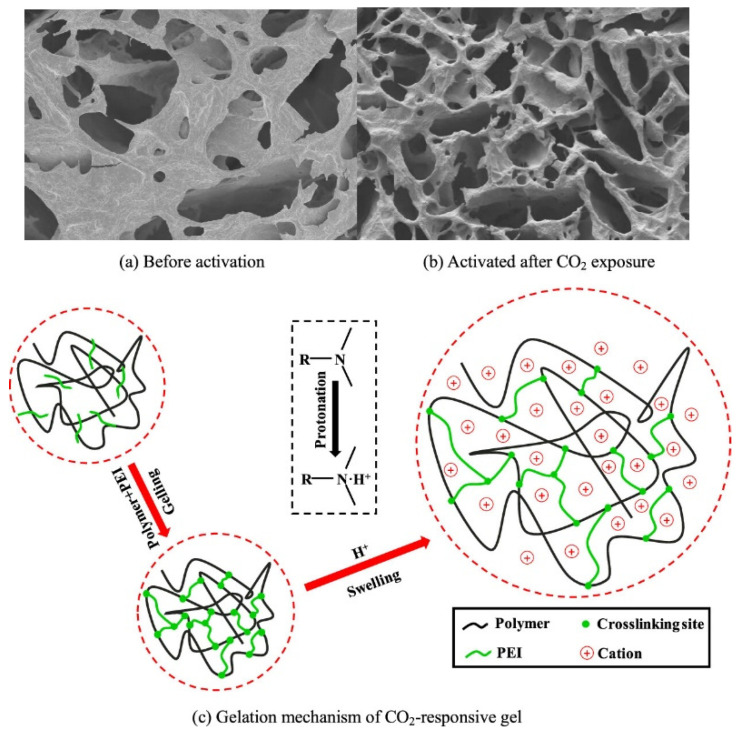
Microscopic structure of the CO_2_ responsive polymer system for CO_2_ capture. (**a**) Before activation; (**b**) Activated after CO_2_ exposure; (**c**) Gelation mechanism of CO_2_-response gel [82].

A novel intelligent polymer sealant has been designed to reduce CO_2_ leakage in underground geological storage [82]. This sealant was generated through the cross-linking of CO_2_-responsive polymers, particularly acrylamide (AM) and N-[3-(dimethylamino) propyl] methacrylamide (DMAPMA), using polyethyleneimine (PEI) as the cross-linking agent. When exposed to CO_2_, the polymer system transitions from a sol to a gel state, resulting in a smooth surface and a uniformly porous three-dimensional network structure that effectively seals potential leakage pathways. The practical efficiency of these systems has been shown by core fluid displacement experiments, revealing a sealing efficiency of 73.6% for CO_2_ and a subsequent injection water sealing rate of 96.2%. The gelation mechanism signifies the binding of CO_2_ to water molecules, leading to the dissociation of H^+^ in aqueous solutions. This process generates an acidic environment that facilitates the attachment of tertiary amine groups to H^+^, thereby converting the gel from a nonionic to a cationic state (Figure 14). This transformation enhances hydrophilicity and increases electrostatic repulsion among polymer segments.

### 4.3. Polymer-Assisted CO_2_ Reduction

**Figure 15:**
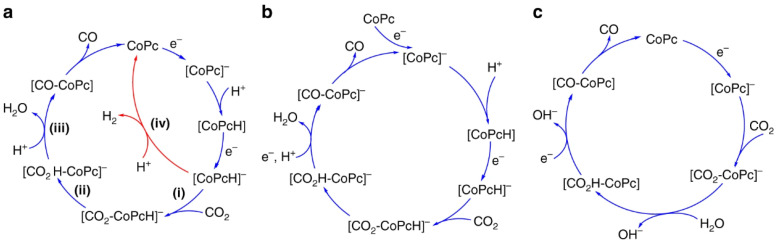
Proposed CO_2_ reduction mechanisms of cobalt phthalocyanine (CoPc). (**a**) A mechanism showing sequential steps for competitive H_2_ generation: (**i**) first reduction of CoPc, (**ii**) protonation forming [CoPcH]^−^, (**iii**) CO_2_ binding to the reduced complex, and (**iv**) CO_2_ reduction to CO or competitive protonation leading to H_2_ generation. (**b**) Alternative mechanism proposed in organic solutions. (**c**) Mechanism proposed in low-concentration bicarbonate buffer under aqueous conditions [136].

Recent advancements indicate that the reduction of CO_2_ can be markedly improved by integrating molecular electrocatalysts with CO_2_-responsive polymer matrices [132,133,134]. A well-researched system includes the encapsulation of cobalt phthalocyanine (CoPc) within poly(4-vinylpyridine) (P4VP), demonstrating significant activity and selectivity for the electrochemical reduction of CO_2_ to carbon monoxide. This enhancement results from a synergistic interaction between the metal complex and the polymer microenvironment, influencing both the thermodynamics and kinetics of the catalytic cycle [135]. Figure 15 illustrates that the proposed mechanism initiates with the stepwise reduction of CoPc, succeeded by the protonation of the complex. In the presence of CO_2_, the reduced and protonated intermediate [CoPcH]^−^ coordinates with CO_2_ to form a [CO_2_-CoPcH]^−^ adduct. Following proton-coupled electron transfer, carbon monoxide is produced and the catalyst is regenerated. In the absence of CO_2_ coordination, an alternative pathway leads to H_2_ evolution via direct protonation. The P4VP polymer affects this mechanism through three primary effects: (1) axial coordination of pyridyl groups to the cobalt center increases CO_2_ binding affinity, (2) hydrogen bonding in the secondary coordination sphere stabilizes reactive intermediates, and (3) proton relays created by protonated pyridyl residues facilitate efficient proton delivery to the active site, which is essential for the rate-determining step. The interactions collectively change the rate-determining step from CO_2_ binding, as seen in bare CoPc systems, to the protonation of the coordinated intermediate in polymer-bound CoPc [136]. The observed shift has been validated through kinetic isotope effect (KIE) and proton inventory studies, indicating a significant reduction in KIE values when P4VP is present, aligning with polymer-mediated proton transfer mechanisms. The findings establish a mechanistic basis for the systematic design of multifunctional CO_2_-responsive polymers capable of both CO_2_ capture and its conversion under mild electrochemical conditions.

### 4.4. Drug Delivery System

**Figure 16:**
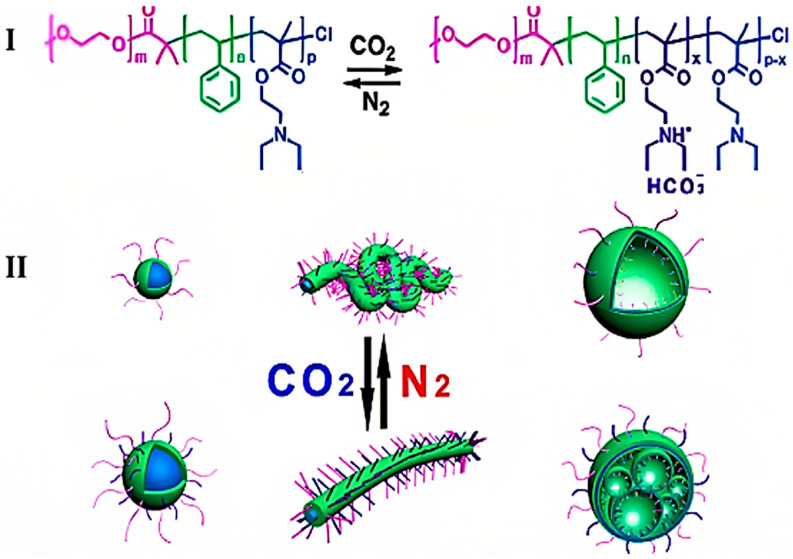
CO_2_-switchable triblock copolymer (**I**) and CO_2_-driven controlled deformation of nanostructures (**II**) [98].

CO_2_-responsive polymers have transformed drug delivery systems by allowing for controlled release mechanisms that are triggered by stimuli. An exemplary case is the fabrication of CO_2_-responsive core–shell–corona structured magnetic Fe_3_O_4_@SiO_2_-poly(N,N-dimethylaminoethyl methacrylate) (PDMAEMA) nanocarriers. The hybrid magnetic nanoparticles exhibit a sandwich structure characterized by superparamagnetic properties and gas-responsive behavior, facilitating controlled drug release. Exposure to CO_2_ induces a change in the hydrodynamic radius of these nanoparticles via a switchable volume transition, leading to the release of encapsulated drugs such as doxorubicin [137]. CO_2_-responsive polymers, such as the amidine-containing block copolymer developed by Yuan et al. [96] can generate vesicles that exhibit a biomimetic “breathing” characteristic in response to CO_2_. This provides a modifiable membrane permeability in reaction to CO_2_ stimulation. Polymers such as poly((N,N-dimethylamino)ethyl methacrylate) and poly((N,N-diethylamino)ethyl methacrylate), which contain tertiary amine groups (Figure 16I), exhibit carbon dioxide responsiveness. This was demonstrated by Zhao et al. [98] through the design of triblock copolymers that can mimic gas-controllable organelle deformations (Figure 16II).

Functionalities that demonstrate responsiveness to CO_2_ include tertiary amines, amidines, and guanidines, which can undergo reversible protonation and deprotonation without generating side products. CO_2_-responsive polymers are garnering considerably more interest in the field of drug delivery compared to polymers responsive to other gas transmitters [31]. Increased CO_2_ levels in the body, referred to as hypercapnia, result in respiratory acidosis due to the excessive production of protons. The reduction in pH may provide advantages for patients with acute lung injury; however, the negative consequences significantly surpass the potential benefits [138]. Tian et al. recently investigated (2-diethylamino)ethyl methacrylate to develop a dual CO_2_- and photo-responsive system, implementing the reversible and dynamic characteristics of CO_2_-responsive systems. This system provided adaptability in volume and wall thickness for the delivery of curcumin. Significant changes in morphology were observed as the self-assemblies underwent different levels of protonation, resulting in a spectrum of morphologies including micelles, worm-like micelles, and small vesicles, corresponding to alterations in the hydrophilic fractions of the nanoaggregates. The protonation of tertiary amines by CO_2_, coupled with the dissociation of anthracene photodimers via UV light, supported the release of curcumin, which could be used for improved localization through external stimuli. This formulation demonstrates a responsive behavior to low pH, with accelerated drug release observed at pH 5.0 in comparison to pH 6.0 and pH 7.4 [139]. In conclusion, although the existing literature offers limited attention to this topic, it highlights considerable potential for diverse applications.

### 4.5. Other Applications

In addition to the previously discussed categories, a variety of innovative applications have recently emerged that harness CO_2_-responsive polymers, owing to the environmentally friendly nature of CO_2_ as a trigger. These applications span multiple fields, including forward osmosis desalination, tissue engineering, smart emulsions, extraction systems, and CO_2_ sensing technologies.

#### 4.5.1. Forward Osmosis Desalination

CO_2_-responsive polymers have demonstrated significant potential in forward osmosis (FO) desalination [46]. Forward osmosis (FO) desalination involves the extraction of clean water from seawater or wastewater via a semipermeable membrane into a concentrated draw solution. An optimal draw solute should generate significant osmotic pressure while being easily removable, non-toxic, and stable [140,141]. A patent introduced CO_2_-switchable polymers as draw solutes, using the bicarbonate salt of a CO_2_-responsive polymer dissolved in carbonated water as the draw solution. Following water transport through osmosis, the elimination of CO_2_ through air or nitrogen bubbling results in polymer precipitation (if insoluble) or facilitates recovery through low-energy reverse osmosis (if soluble), producing fresh water [142]. Jessop and colleagues proposed the application of PDEAEMA, which, following neutralization, exhibits water-insolubility and is easily recoverable [143]. PDMAEMA is used as a draw solute, with significant water flux in forward osmosis. Following CO_2_ removal and moderate heating, PDMAEMA precipitates, providing straightforward separation while minimizing membrane degradation, back-diffusion, and toxicity [106].

Beyond water treatment applications, CO_2_-responsive polymers have been investigated for use in smart ion-channel devices. Jiang et al. [144] synthesized a CO_2_-responsive ion channel that is coated with PDEAEMA polymer brushes at both openings. These polymer films exhibit reversible transitions between hydrophobic and hydrophilic states in response to the addition or removal of CO_2_, providing voltage-independent regulation of their open and closed configurations. These systems exhibit potential applications in energy, filtration, and seawater desalination.

#### 4.5.2. Tissue Engineering

In tissue engineering, CO_2_ serves as an important agent for polymer processing, particularly in the fabrication of scaffolds. Since the mid-1990s, CO_2_ has been used for the production of polymeric foams for applications in regenerative medicine [47,145]. Researchers have systematically optimized parameters to enhance pore interconnectivity and scaffold performance [145]. In addition to foaming, CO_2_ serves as a swelling agent for loading scaffolds with bioactive compounds and encapsulating plasmids for gene delivery. The application of low-pressure CO_2_ sintering for polymeric microspheres embedded with living cells represents a significant advancement, facilitating the one-step production of scaffolds devoid of organic solvents. This innovation enhances usability, reduces costs, and improves compatibility with polymers such as PLA, PGA, PEG, and PCL, thereby significantly broadening the scope of tissue engineering applications [146].

#### 4.5.3. Smart Emulsions

Last but not least, CO_2_-responsive polymers have made significant advances in emulsion technologies. Chitosan-g-poly[(2-dimethylamino)ethyl methacrylate] has been developed as a CO_2_-switchable emulsifier, demonstrating reversible emulsification that is responsive to temperature and pH through the alternating bubbling of CO_2_ and N_2_. Stable emulsions of n-butanol and water can be generated through CO_2_ bubbling and disrupted by N_2_ bubbling. The development of two-way CO_2_-responsive polymer particles that use PMAA-b-PDMAEMA diblock copolymers represents an important milestone [147]. These particles promote reversible aggregation and dispersion across different pH levels, providing “green emulsion” technologies with programmable amphiphilic characteristics, which are beneficial for industries requiring precise emulsification and separation processes. CO_2_-responsive materials in analytical chemistry contributed to more environmentally friendly solid-phase extraction (SPE) techniques. Researchers have shown that CO_2_-responsive silica can effectively pre-concentrate analytes using solely water and carbonated water, thereby reducing the reliance on organic solvents and promoting sustainability, all while preserving high separation efficiency [60,148].

#### 4.5.4. CO_2_ Sensing

Finally, CO_2_-responsive polymers are becoming more prevalent in CO_2_ sensing applications. Kang’s group designed a fluorophore-functionalized PDMAEMA polymer that exhibits reversible color changes resulting from the protonation or deprotonation of amine groups in response to the addition or removal of CO_2_ [99]. The solution transitions from dark red to orange, offering CO_2_ detection in aqueous solutions. Yung’s group developed an intuitive detection system by combining gold nanoparticles with a random copolymer (P(DMA-co-NAEAA)) that contains amidine groups. The dissolution of CO_2_ resulted in the protonation of amidine groups, leading to nanoparticle aggregation through electrostatic interactions, which manifested in plasmonic changes observable by the naked eye or through UV–Vis spectroscopy [149]. Additionally, Guo’s group developed CO_2_-responsive carbon nanotube (CNT) sensors that can detect dissolved CO_2_ in water, representing another significant application of CO_2_-responsive materials [150].

## 5. Challenges and Future Perspectives

CO_2_-responsive polymers represent a significant category of smart materials, characterized by their capacity to reversibly change physicochemical properties upon exposure to CO_2_. These polymers, which generally contain functional groups like amidines, amines, or carboxyls, present unique advantages compared to conventional stimuli-responsive materials, particularly through the use of CO_2_ as a safe, plentiful, cost-effective, and eco-friendly trigger [28,32,86]. This review has systematically investigated the fundamental mechanisms underlying the responsiveness of CO_2_-responsive polymers, with a particular emphasis on recent advancements in synthetic strategies and their expanding range of latest applications. These materials have been successfully applied across diverse fields, including oil–water separation, carbon capture and storage, drug delivery, tissue engineering, forward osmosis desalination, smart emulsions, extraction systems, and CO_2_ sensing. Their unique ability to reversibly switch between hydrophilic and hydrophobic states, combined with self-cleaning capabilities and operation under mild conditions, makes them highly promising candidates for the development of sustainable and energy-efficient technologies.

Despite these promising developments, several unresolved challenges must be addressed to advance the practical deployment of CO_2_-responsive polymers. First, precise control over switching kinetics and reversibility remains a major obstacle; rapid, low-energy responsiveness under fluctuating conditions is essential for real-time and long-term performance. Cyclic stability is another concern—many polymers experience mechanical degradation, fouling, or loss of activity after repeated switching cycles, particularly in membrane and separation technologies.

In biomedical applications such as drug delivery, achieving spatial and temporal control over CO_2_ levels in vivo is difficult and raises critical issues surrounding biocompatibility, degradation products, and off-target effects. Similarly, in forward osmosis and purification systems, challenges include the high viscosity of polymer solutions, membrane fouling, and limited recyclability. Environmental variables, such as temperature, humidity, pH, and coexisting species, can also interfere with polymer response, especially in real-world sensing or extraction contexts. Additionally, the tuning of thermal transitions (Tg, LCST) remains crucial to prevent unwanted aggregation or inconsistent behavior across applications.

Moving forward, a major research priority lies in developing scalable and environmentally sustainable synthesis strategies for CO_2_-responsive polymers. Future efforts should focus on refining controlled/living radical polymerization and adopting green chemistry approaches to enable low-cost, large-scale production. Designing polymers with enhanced architecture, such as block copolymers, grafts, or hyperbranched structures, can improve responsiveness, speed, and durability. Special emphasis should be placed on creating biodegradable or recyclable CO_2_-responsive systems, especially for biomedical and environmental applications.

The integration of computational modeling and machine learning can significantly accelerate the design of next-generation materials by predicting structure–function relationships and optimizing polymer formulations. Exploring hybrid materials, such as polymer–inorganic composites or multi-stimuli-responsive systems, may also yield synergistic properties suitable for complex operating environments. Furthermore, the industrial integration of CO_2_-responsive materials into continuous flow systems, programmable membranes, or CO_2_ capture platforms remains an open and important avenue for translation from laboratory innovation to large-scale impact. By addressing these open questions and interdisciplinary challenges, CO_2_-responsive polymers have the potential to drive sustainable innovations in energy, water, healthcare, and environmental remediation.

## Figures and Tables

**Table 1:** CO_2_-responsive polymers synthesized via RAFT polymerization.

Method Name	CO_2_-Responsive Polymer Type	Key Findings	References
RAFT Polymerization	PDEAEMA-b-PNIPAM (block copolymer)	Dual CO_2_- and temperature-responsive block copolymer synthesized via RAFT. Shows switchable solubility and hydrophilicity under CO_2_.	[106]
RAFT Polymerization	Poly(4-chloromethylstyrene)—PCO_2_-switchable amidine-based polymer	PCMS prepared by RAFT, then converted to PAMS via click chemistry, followed by amidine functionalization, resulting in CO_2_-responsive behavior.	[107]
RAFT Polymerization	CO_2_-switchable single-walled carbon nanotubes (SWCNTs)	SWCNTs were functionalized with CO_2_-responsive polymeric brushes using RAFT polymerization for improved environmental adaptability and selectivity under CO_2_.	[29]
RAFT Polymerization	PEO-b-DEAEMA-r-S and PEO-b-DEAEMA-b-S (diblock copolymers)	PEO-based block copolymers with CO_2_-switchable units (DEAEMA) designed for morphological transitions in aqueous solution.	[108]
RAFT Polymerization	PEO-b-(PDEAEMA-r-S) (triblock copolymer)	CO_2_-responsive triblock copolymer with segregated corona synthesized by sequential RAFT, showcasing morphological transitions on CO_2_ bubbling.	[109]
RAFT Polymerization	Poly(pentafluorophenyl acrylate)—PDMAEMA and PEGA block copolymers	Dual-responsive copolymers synthesized by RAFT, functionalized with histamine and arginine to achieve CO_2_-switchability.	[54]
RAFT Polymerization	PMMA-b-PDMAEMA (diblock copolymer)	CO_2_-responsive PMMA-b-PDMAEMA block copolymer synthesized via RAFT. Demonstrated self-assembly behavior in aqueous media under CO_2_–N_2_ cycling.	[110]
RAFT Polymerization	PNIPAM-b-PCL-b-PDMAEMA (triblock copolymer)	Dual-responsive PNIPAM-b-PCL-b-PDMAEMA copolymer synthesized by RAFT, responsive to temperature and CO_2_.	[111]
RAFT Polymerization	Poly(oligo(ethylene glycol)methyl ether methacrylate)-b-PDEAEMA-b-PAPUEMA	CO_2_- and temperature-responsive triblock copolymer synthesized by sequential RAFT polymerization for responsive polymeric brushes.	[112]
RAFT Polymerization	CO_2_-switchable pyrene-containing copolymer (Py-PCL-b-P(NIPAM-co-DMAEMA))	Amphiphilic copolymer synthesized by RAFT for micelle formation in response to CO_2_.	[113]
RAFT Polymerization	CO_2_-responsive polystyrene (PS)-PDEAEMA nanoparticles	Nanoparticles prepared via surfactant-free mini-emulsion RAFT polymerization. Exhibited reversible self-assembly in response to CO_2_.	[114]

**Table 2:** Comparative analysis of polymerization methods for CO_2_-responsive polymers.

Polymerization Method	Key Advantages	Key Disadvantages
Free radical polymerization (FRP)	Economical and straightforward Commonly utilized and capable of scaling Suitable for high molecular weight polymer	Restricted control over molecular weight and dispersity Inability to synthesize block copolymers with narrow dispersity
Reversible deactivation radical polymerization (RDRP)	Offers accurate control of molecular weight Facilitates the synthesis of block copolymers Low dispersity (Đ < 1.5)	More complex setup than FRP Sensitive to oxygen and moisture in some cases
Atom transfer radical polymerization (ATRP)	Exceptional control over molecular weight and architecture Compatible with a variety of monomers, including CO_2_-responsive functional monomers	Must require a metal catalyst (e.g., copper) Prone to oxidation; removing catalysts can be challenging
Reversible addition-fragmentation chain transfer (RAFT)	Adaptable for various monomers Functions effectively in aqueous environments Facilitates the creation of precisely defined block copolymers with minimal dispersity	Possibility of color variation in the finished polymer Removal of post-polymerization RAFT agent is necessary
Nitroxide-mediated polymerization (NMP)	Process without metal Easy and affordable Perfect for monomers with amine functionality	Demands high temperatures for activation Monomer compatibility is more restricted in comparison to RAFT or ATRP

**Table 3:** Overview of CO_2_-responsive polymers in oil–water separation applications.

Polymer/Material	CO_2_ Stimulus	Method of CO_2_-Responsive Polymer Preparation	Oil–Water Separation Key Findings	Reference
Poly(styrene-co-N,N-(diethylamino)ethyl methacrylate)	CO_2_ switches wettability from hydrophobic–superoleophilic to hydrophilic–superoleophobic	Free radical polymerization (FRP)	Efficient and recyclable switchable oil–water separation driven by gravity	[124]
PDEAEMA polymer on PU sponge	CO_2_ induces protonation, switching from hydrophobic to hydrophilic	Free radical polymerization (FRP)	Demonstrates a high oil adsorption capacity of 14 g/g for emulsified oil, achieving a separation efficiency of 97.5%	[126]
Polymerized PDEAEMA on CNFs aerogels	CO_2_ induces protonation in PDEAEMA, altering wettability between hydrophobic and hydrophilic	Surface-Initiated Atom-Transfer Radical Polymerization (SI-ATRP)	Achieves oil–water separation efficiency of up to 99.96%	[127]
CO_2_-responsive cellulose nanofibers aerogels	CO_2_-induced wettability change from hydrophobic to hydrophilic for oil–water separation	Surface-Initiated Atom-Transfer Radical Polymerization (SI-ATRP)	Cellulose aerogels that are recyclable and have switchable wettability for the separation of oil–water mixtures	[128]
CO_2_-responsive nanofibrous membranes	CO_2_ causes a switchable hydrophilic to hydrophobic transition in nanofibers	Electrospinning (free radical polymerization)	Efficient selective separation of oil and water deploying switchable wettability	[129]

## Data Availability

Not applicable.
